# N^6^-Methyladenosine Regulator-Mediated RNA Methylation Is Involved in Primary Sjögren's Syndrome Immunoinfiltration

**DOI:** 10.1155/2022/5242287

**Published:** 2022-11-22

**Authors:** Fang He, Hexu Liu, Changyin Yu

**Affiliations:** ^1^Key Laboratory of Cell Engineering of Guizhou Province, Affiliated Hospital of Zunyi Medical University, Zunyi, China; ^2^Department of Neurology, Affiliated Hospital of Zunyi Medical University, Zunyi, China

## Abstract

The crucial role of epigenetic regulation, especially the modifications of RNA N^6^-methyladenosine (m6A), in immunity is a current research hotspot. However, the m6A modifications in primary Sjögren's syndrome (pSS) and the immune infiltration pattern they govern remain unknown. Thus, the patterns of 23 m6A regulator-mediated RNA modifications in parotid or blood samples from pSS patients were evaluated by bioinformatics analysis in the current study. Comparing m6A regulators between control and pSS patients showed that m6A regulators are associated with pSS, and regulators also had differential correlations. Further clustering analysis and comparison of gene expression and immune cell infiltration between m6A modification patterns revealed that each modification pattern had its own unique genetic and immune profile. Multiple immune cell infiltrations were differentially expressed between the patterns. The enrichment of gene ontology between the two patterns in parotid was concentrated on RNA metabolism and processing. The KEGG pathway enrichment and weighted correlation network analysis further showed that the autophagy pathway might be involved in the m6A modification patterns in pSS. Together, these findings suggest that m6A regulators play a certain role in the immune cell infiltration of parotid tissue in pSS.

## 1. Introduction

Primary Sjögren's syndrome (pSS) causes significant damage to the exocrine glands, resulting in decreased lacrimal and salivary production [1]. Dry mouth caused by salivary gland dysfunction may further lead to a range of oral diseases such as tooth decay, salivary gland inflammation, altered taste, bad breath, and painful swallowing. In addition to the characteristic glandular symptoms, other systemic symptoms, like extra-glandular manifestations, are also seen in a small percentage of individuals [2]. Numerous studies have shown that immune cell infiltration is a prominent pathological feature of pSS [3, 4]. For example, the number of CD4^+^CD25^+^ Treg cells and FoxP3 protein expression are significantly reduced in salivary gland biopsies from patients with pSS compared to healthy individuals, and the FoxP3 expression in peripheral blood is similarly reduced in this patient population [5–7]. Routinely, the clinical diagnosis of pSS is usually based on the presence of dry eyes and dry mouth symptoms, usually with objective evidence of keratoconjunctivitis and/or reduced salivary flow, and the widely accepted histological criteria for confirming pSS is massive immune cell infiltration of the salivary glands [8]. From a pathological point of view, the elimination of inflammatory infiltrates and reversal of glandular dysfunction remain significant challenges in the clinical management of this disease. Hence, understanding the immune-related mechanisms of pSS may be the key to uncovering its pathology and may reveal some new immunotherapeutic approaches for pSS, which may also further alleviate the development of oral disease complications.

Traditionally, epigenetics includes reversible modifications of DNA and histones, which can be expressed independently of DNA sequences for genes [9]. Recently, RNA modifications, such as RNA processing and metabolism, have been considered a new layer of epigenetic phenomena [10, 11]. RNA modifications are present in all organisms, and more than 150 modifications have been identified, the most abundant form of which is RNA N^6^-methyladenosine (m6A) [12, 13]. m6A modifications are dynamic in eukaryotic cells mediated by a combination of regulation by methyltransferases, demethylases and binding proteins, which are cited as “writers”, “erasers”, and “readers”, respectively [14]. Specifically, the methylation process of m6A is regulated by methyltransferases, including METTL3, METTL14, WTAP, ZC3H13, RBM15, RBM15B, CBLL1, and KIAA1429; demethylases control the demethylation of m6A, including ALKBH5 and FTO; and m6A readers are proteins that bind to m6A to recognize its methylation pattern, and subsequently mediate the regulatory function of m6A, such as the YTHDF and IGF2BP families [15].

m6A regulation can explain some of the basic mechanisms of immune regulation of systemic autoimmune diseases [16]. However, the role of m6A in the pathogenesis of pSS, particularly the immune cell-mediated response in the parotid tissue of patients, has not been reported. In this study, a detailed examination of the immune cell's alterations among the various subtypes of m6A modification patterns of pSS, as well as the elaboration of the biological phenomena mediated between the patterns were performed by bioinformatics analysis. These findings will help researchers to better grasp the pathophysiology of pSS and may shed light on the pathogenesis of pSS from a novel perspective, potentially revealing some new potential targets for treating pSS.

## 2. Method

### 2.1. Data Processing

The data for this study came from two different sample sets: parotid tissue and whole blood. The parotid tissue study for pSS encompassed 18 control samples (patients without subjective symptoms of dry mouth and dry eyes) and 17 pSS samples. The whole blood sample study for pSS included 30 healthy control samples and 30 pSS samples. Sample handling methods and RNA extraction protocols have been described in previous studies [17, 18]. Affymetrix Human Genome U133 Plus 2.0 Array chips were used to measure gene expression in the samples according to the manufacturer's recommendations. The serial numbers GSE40611 for parotid samples and GSE84844 for blood samples are stored in the GEO (Gene expression omnibus) database. The acquired data were normalized with the “normalizeBetweenArrays” order in the R software package “limma” [19]. The R-4.1.1-win version of the R software was used in the current study [20].

### 2.2. Analysis of Alterations in m6A Regulators between Control and pSS in Parotid and Blood Samples

The 23 m6A regulators' protein-protein interaction networks were reviewed from the metascape database (https://metascape.org) [21]. To discover densely coupled network components and define their roles, the Molecular Complex Detection (MCODE) method was used [22]. For m6A studies in pSS, the expression of m6A regulator-related genes was obtained from parotid and whole blood datasets and used to study the expression relationships of 23 m6A regulators in control and pSS groups. The differences in expression status of the 23 m6A regulators between the two groups were compared by Wilcoxon-Vorzeichen-Rang-Test. The R packages “heat map”, “reshape2”, and “ggpubr” were used for the illustration of heatmaps and box plots. Additionally, Spearman correlation analysis was used to examine the correlation between “readers” and “erasers”.

### 2.3. Identification of m6A Modification Pattern in pSS

A cluster analysis was performed on pSS samples to identify different m6A modification patterns based on the expression of 23 m6A regulators. To determine the number and strength of clusters, consensus clustering techniques based on the R package “ConsensusClusterPlus” were employed. Based on the data of consensus clustering and the principal component analysis (PCA), we further validated the expression of m6A regulators in different modification patterns. The m6A regulators' expressions were compared between the two (blood and parotid) modification patterns.

### 2.4. Analysis of Immune Cell Infiltration in Different m6A Modification Patterns

A single sample gene set enrichment analysis (ssGSEA) [23] was applied to estimate the specific immune cell populations, and an assessment of the activity of specific immune responses infiltrated into different m6A modification patterns was conducted. Further, the correlation of m6A modulators with immune cell fractions was determined by spearman correlation analysis. Based on the comparison results of m6A regulators-related gene expression between the two patterns, the two genes with the relatively highest significant differences in the blood (FMR1 and IGF2BP2) and the parotid (HNRNPC and FMR1) samples were selected for further analysis. The samples were divided into two groups of high and low expression according to the expression of the target genes, and the immune cell infiltration was compared between the two groups.

### 2.5. Biological Enrichment Analysis for the m6A Modification Pattern in Parotids

In response to the biological phenomena regulated by the m6A modification pattern in the parotid gland, differentially expressed genes of the two m6A modification patterns were used for gene ontology (biological processes, molecular function, and cellular components) and KEGG pathway enrichment analysis. Analysis of variance was set to adjust for an adjusted *p* value of <0.05 as a cut-off criterion. “enrichGO” and “enrichKEGG” orders were performed in R software for gene ontology and pathway enrichment analysis. Circle charts, bar charts, and bubble charts are used for the result exhibition.

### 2.6. Identification of Genes Mediated by m6A Regulators by Weighted Gene Co-Expression Network Analysis (WGCNA)

The R package “WGCNA” was loaded and used to construct a network of coexpression modules and to identify genes mediated by the m6A regulator [24, 25]. In brief, after constructing the coexpression similarity and the family of adjacency functions, the adjacency matrix of genes is converted into a topological overlap matrix (TOM) matrix, and then the color modules are determined by the TOM-based dissimilarity and similar module clustering. Finally, relationships between m6A modification patterns and color modules were determined. The bubble chart of the module membership in the optimal module versus the gene significance was illustrated. The top 100 interactions of the nodes in the optimal module based on the weight rank were analyzed and illustrated by Cytoscape software. In addition, KEGG signaling pathway enrichment of optimal module genes was performed, and the top 10 genes in the optimal module were analyzed by the network string_interactions (https://string-db.org/) [26] and Cytoscape software [27].

## 3. Results

### 3.1. The Landscape of m6A Regulators

**Figure 1:**
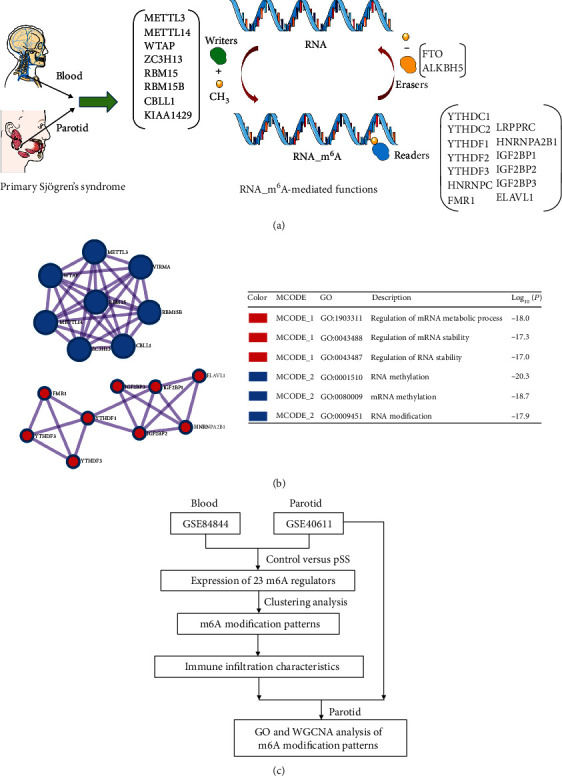
The expression profile of the m6A RNA methylation regulators in pSS is shown. (a) The m6A RNA methylation modifications of “writers”, “erasers” and “readers” govern the dynamic reversible biological processes in pSS. (b) Protein-protein interactions for the 23 m6A RNA methylation regulators were investigated. (c) Research flow chart for this study.

This investigation involves 23 m6A regulators, comprising 8 writers, 13 readers, and 2 erasers.  Figure 1(a) summarizes the functional profile of m6A regulators in blood and parotid samples of pSS patients. The protein-protein interaction enrichment analysis of the 23 m6A regulators has been carried out in the Metascape platform based on the Molecular Complex Detection (MCODE) algorithm (Figure 1(b)). The regulatory interactions of m6A can be classified into MCODE_1 and MCODE_2 subtypes. The MCODE_1, which is centered on the RBM15, is described as the regulation of the mRNA metabolic process and the regulation of mRNA/RNA stability. Meanwhile, MCODE_2 comprises RNA/mRNA methylation and RNA modification. In addition, the research flow chart for this study is shown in Figure 1(c).

### 3.2. m6A Regulators Are Involved in pSS

**Figure 2:**
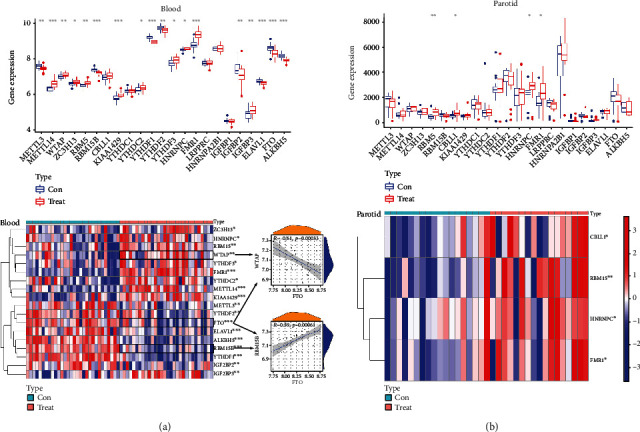
The transcriptome expression status of 23 m6A regulators in the blood (a) and the parotid (b) was compared between control (con) and Sjögren's syndrome (treat) using a box plot and a heat map plot.

To investigate the possible contribution of m6A to pSS, we compared the gene expression of 23 m6A regulators in normal control (con) and patient (treat) groups. As far as the blood samples were concerned, the box plot and heat map showed that except for the genes CBLL1, YTHDC1, LRPPRC, HNRNPA2B1 and IGF2BP1, other genes in the 23 m6A regulators were up- or down-regulated between the two groups (Figure 2(a)). In addition, correlation analysis between “erasers” and “writers” regulators showed a negative correlation between FTO and WTAP and a positive correlation between FTO and RBM15B in the blood samples of pSS. The box plot and heat map for the parotid samples showed that RBM15, CBLL1, HNRNPC, and FMR1 genes possess different expressions between control and patient groups (Figure 2(b)).

### 3.3. The m6A Modification Patterns Mediated by 23 Regulators in pSS

**Figure 3:**
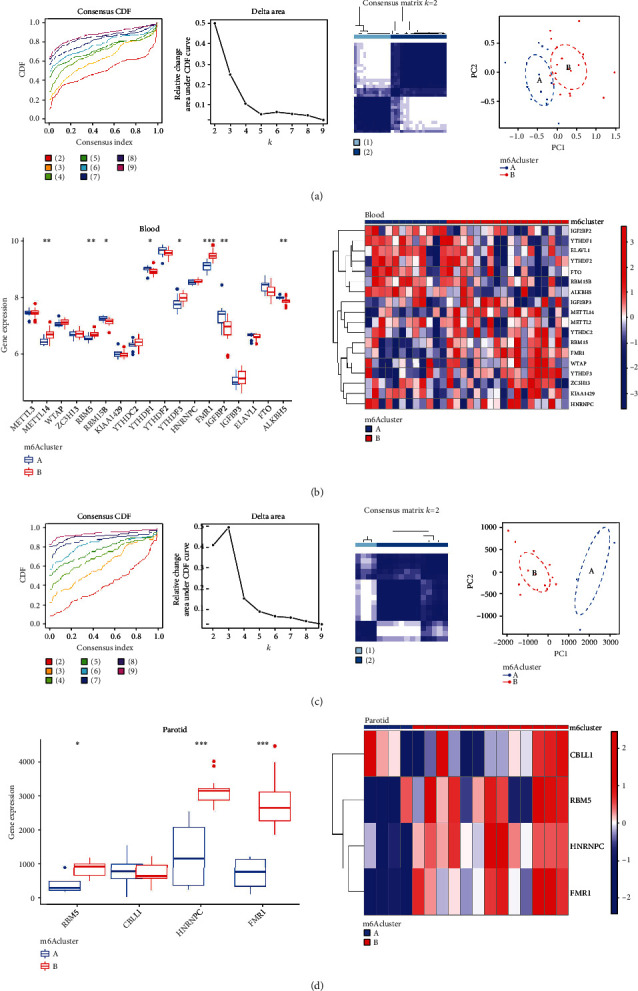
Consensus clustering of 23 m6A regulators for blood or parotid samples in Sjögren's syndrome. (a) The cumulative distribution function (CDF), the relative change in area under the CDF curve, the heat map of the consensus matrix and the principal component analysis (PCA) for blood samples in pSS. (b) The expression status of m6A regulators between the two m6A subtypes of modification patterns in blood samples of pSS is shown as a box plot and a heat map plot. (c) CDF, the relative change in area under the CDF curve, the heat map of the consensus matrix and the PCA for the parotid samples in pSS. (d) The expression status of m6A regulators between the two m6A modification patterns in parotid samples of pSS is shown as a box plot and a heat map plot.

In order to better understand m6A modification patterns in pSS samples based on the expression of 23 m6A regulators, we performed a consensus clustering analysis. Two distinct modification patterns of pSS were identified for the blood samples, including 12 samples in subtype-A and 18 in subtype-B (Figure 3(a)). METTL14, RBM15, RBM15B, YTHDF1, YTHDF3, FMR1, IGF2BP2, and ALKBH5 showed a clear difference between the subtypes, with the most notable differences in FMR1 (Figure 3(b)). In addition, two distinct modification patterns of pSS have been identified in parotid samples, including 4 samples belonging to subtype-A and 13 samples belonging to subtype-B (Figure 3(c)). The genes RBM15, HNRNPC, and FMR1 are differently expressed between clusters A and B in parotid samples, with the most notable differences in HNRNPC and FMR1 (Figure 3(d)).

### 3.4. Immune Infiltration Characteristics in Distinct m6A Modification Patterns

**Figure 4:**
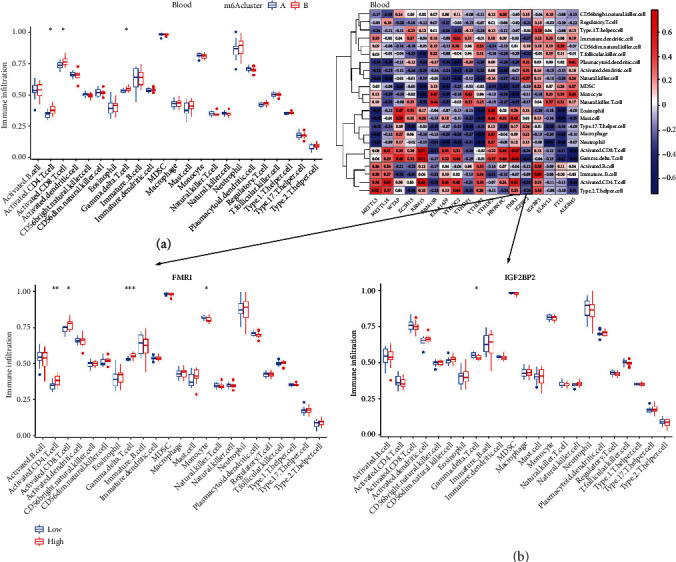
Differential analysis of immune cell infiltration from blood samples in the subtypes of m6A modification patterns. (a) Several differences in immunocytotic infiltration were observed in two m6A modification patterns. (b) The correlation between infiltrating immunocytes and m6A regulators, as well as a comparison of immunocytotic infiltrating between high and low expression groups for FMR1 or IGF2BP2.

To identify immunomodulatory differences between these different m6A modification patterns in blood and parotid samples, we evaluated immune cells infiltrated into different subsets. For the blood samples, Activated CD4 T cell, Activated CD8 T cell, and Gamma-delta T cell were differentially expressed between the two m6A subsets (Figure 4(a)). The correlation analysis heat map shows a varying positive (red) or negative (blue) correlation coefficient between different immune cells and the m6A genes (Figure 4(b)). In particular, RBM15 and Activated CD8 T cell showed the highest positive correlation (value: 0.77). YTHDF2 and Neutrophil, on the other hand, showed the highest negative correlation (value: -0.72). Further, the relationship analysis between Gene MFR1 and immune cells showed the differential infiltration of Activated CD4 T cell, Activated CD8 T cell, Gamma-delta T cell, and Monocyte are found between the two groups with high- and low-MFR1 expression. Gamma-delta T cell was differentially infiltrated between IGF2BP2 low- and high-expression groups.

**Figure 5:**
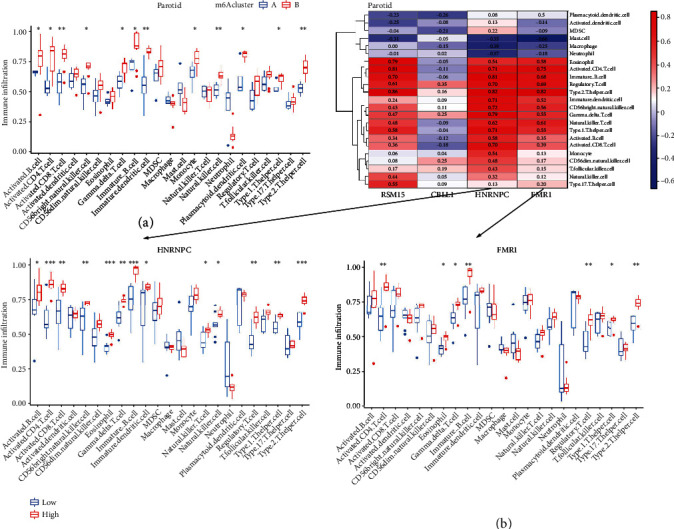
Differential analysis of immune cell infiltration from the parotid gland in the two m6A modification subtypes. (a) In two m6A modification patterns, abundant disparities of immunocytotic infiltrating were observed. (b) The correlation between infiltrating immunocytes and m6A regulators, and the comparison of immunocytotic infiltrating between the high and low expression groups for gene FMR1 or HNRNPC.

For the parotid samples, multiple immune cell infiltration differences appear in subsets A or B (Figure 5(a)). Among them, Activated CD8 T cell, Immature dendritic cell, Natural killer cell, Type 2 T helper cell showed the most significant differences in infiltration. The correlation analysis heat map also shows a varying positive (red) or negative (blue) correlation coefficient between immune cells and the RBM15, CBLL1, HNRNPC, and FMR1 genes (Figure 5(b)). Of these, RBM15 and Type 2 T helper cell showed the highest positive correlation (value: 0.86); FMR1 and mast cell had the highest negative correlation (value: -0.66). Further relationship analysis between HNRNPC/FMR1 and immune cells showed abundant immune cell infiltration in different high- and low-expression groups of the HNRNPC/FMR1 gene.

### 3.5. Biological Activities and Features of m6A Modification Patterns in Parotids

**Figure 6:**
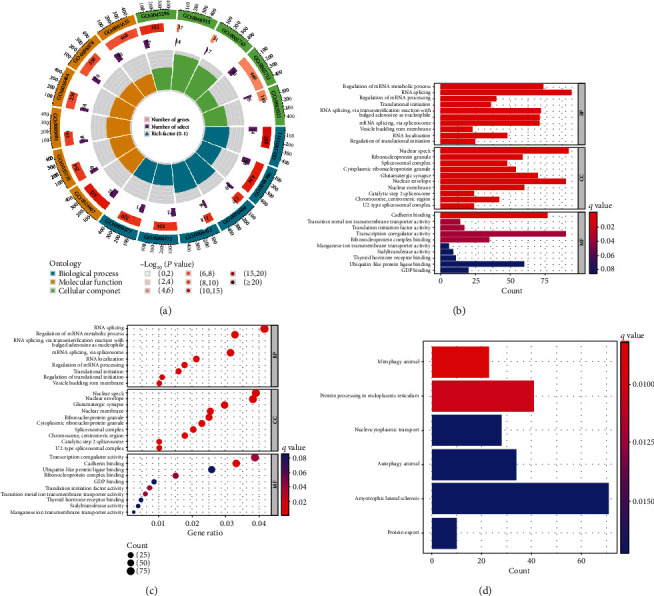
Gene ontology and KEGG pathway enrichment analysis of significant genes associated with m6A modification patterns in the parotid of Sjögren's syndrome (a) The annular chart shows the total number (outer second loop) and the number of significant differences (outer third loop) of genes enriched in biological processes, molecular functions, and cellular components (outer first loop). (b) The barplot of gene ontology enrichment analysis (BP: biological process; CC: cellular component; MF: molecular function). (c) The bubble diagram of gene ontology enrichment. (d) The barplot of KEGG signalling pathway enrichment.

To assess the biological reactions of the m6A modification patterns in pSS, we enriched the biological process, molecular function, and cellular component of ontology in the significantly different genes between the modification patterns A and B in the parotid. A comprehensive gene landscape of Circline showed that 94 obvious different genes in a total of 434 genes were enriched in the biological process GO0008380, 92 obvious different genes in a total of 412 genes were enriched in molecular function GO0016607, and 90 obvious different genes in a total of 489 genes were enriched in cellular component GO0003712 (Figure 6(a)). The barplot and bubble diagram illustrations also showed that the RNA splicing, the nuclear speck and the transcription coregulator activity/the cadherin binding were enriched in BP, CC, and MF, respectively (Figures 6(b) and 6(c)). KEGG signaling pathway enrichment showed that genes associated with m6A modification patterns showed minimal *q* values on the pathway mitophagy-animal (Figure 6(d)).

**Figure 7:**
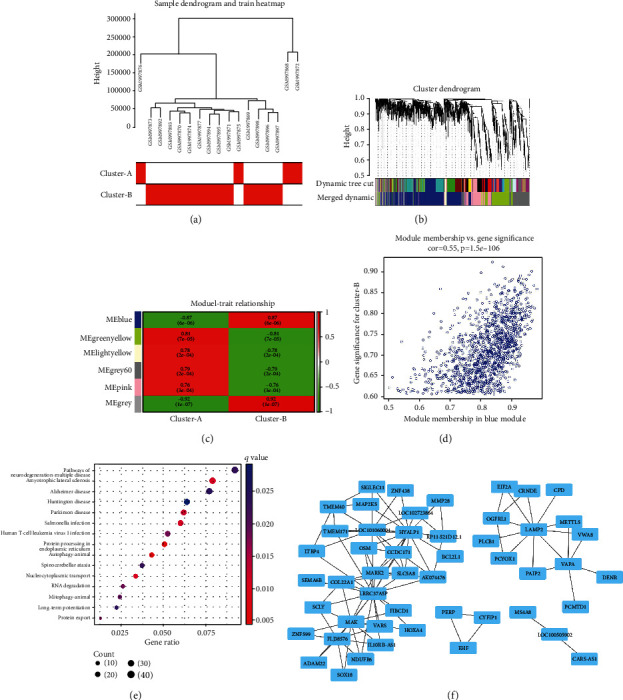
Gene clustering and gene modules related to the m6A-mediated patterns in the parotid of Sjögren's syndrome based on the weighted gene co-expression network analysis (WGCNA). (a) Sample dendrogram and trait heat map based on the m6A modification patterns. (b) The cluster dendrogram based on the heat map of dynamic tree cutting and the merged dynamic. (c) The module-cluster relationships between different color modules and the m6A modification pattern (cluster) A or B. (d) The scatter diagram for the gene significance for cluster B versus the module membership in the blue module. (e) Enrichment of KEGG signalling pathways for genes in the blue module. (f) Based on the weight rank, the top 100 interactions of the nodes in the blue module.

In addition, the sample dendrogram, cluster dendrograms and module-treat relationships identified by WGCNA showed that the blue module presents an obvious negative/positive correlation with m6A modification pattern (cluster) A/B (Figures 7(a), 7(b), 7(c), 7(d)). The scatter plot shows an association value of 0.55 for the module membership in the blue module with the gene significance for m6A regulator modification pattern-B (Figure 7(d)). Meanwhile, KEGG signalling pathway enrichment of blue module genes showed protein processing in the endoplasmic reticulum and autophagy were enriched (Figure 7(e)). Further, the top 100 interactions' map of the nodes ranked by weight in the blue module based on the weight rank showed an intricate network of interactions (Figure 7(f)). Network string_interaction and Cytoscape analysis of all the genes in the blue module showed that MYC, ESR1, HSPA5, EIF4E, PSMA3, EIF2S1, SUMO1, HSPA9, NCBP2 and CYCS are the top 10 critical genes in the blue module.

## 4. Discussion

m6A is the prevalent RNA modification in coding and noncoding RNAs, which may regulate immune cells such as B cells and Tregs to enhance or protect against autoimmune diseases to varying degrees [28–31]. In this study, bioinformatics analysis showed that m6A regulators were involved in pSS, while there were different correlations between regulators. The clustering analysis and immune cell infiltration analysis also showed that each m6A modification pattern has its unique immune profile.

The m6A regulators were differentially expressed in the normal and pSS groups. Among them, WTAP interacts with METTL3 and METTL14 and is required for their recruitment and localization [32], while by interacting with METTL3 in a WTAP-dependent manner, RBM15/15B binds to Uracil enrichment regions and may facilitate the methylation of specific RNAs [33, 34], which has been demonstrated by studies. Therefore, the negative/positive correlations between the eraser FTO gene and the writer WTAP/RBM15B gene in this study suggest that the “eraser” FTO may have potential relevance in the interaction between WTAP/RBM15B and METTL3.

Clustering analysis showed that different m6A modification patterns possess differential gene expressions, with FMR1 being the most significant in blood samples and FMR1 and HNRNPC in parotid samples. These suggest that FMR1 may be the critical differential gene between the different m6A modification patterns in both blood and parotid samples of pSS. FMR1 contains three KH structural domains and one RGG structural domain, which has the potential to influence RNA transfer and stabilization by interacting with YTHDF1 and YTHDF2 [35, 36]. Further, we also observed that the YTHDF1 gene was differentially expressed between different m6A modification patterns. Therefore, the binding of FMR1 and YTHDF1 mediated by m6A modification patterns can be further investigated, which may contribute to understanding the m6A bonding mode in pSS development. In addition, differential infiltration of different immune cells in the high and low expression groups of the FMR1 gene (blood) or HNRNP/FMR1 gene (parotid) also confirmed that the FMR1 regulator is acting as an immune modifier in pSS.

In immune infiltration analysis, each modification pattern also has its unique immune profile. Correlation analysis of immune cells and genes showed the highest negative correlation for YTHDF2/neutrophil in blood samples; consistent with one study that found METTL3 cooperating with YTHDF2 can inhibit papillary thyroid cancer progression via m6A/c-Rel/IL-8-mediated neutrophil infiltration [37]. Interestingly, mast cells can induce tissue fibrosis in developing pSS [38, 39]. The correlation between mast cell and FMR1 showed the highest negative value, suggesting an m6A regulator-FMR1 mediated parotid tissue inflammatory fibrosis remission in pSS.

Mitochondrial dysfunction is involved in pSS. A study has shown that there is a close correlation between mitochondrial dysfunction and the immune microenvironment of salivary glands in patients with pSS [40]. In our study, KEGG signaling pathway enrichment of the differential gene between two m6A modification patterns is enriched in mitochondrial autophagy, which suggests m6A may affect pSS by mediating mitochondrial autophagy. In addition, WGCNA identified genes of the blue synthesis module were enriched for protein processing in the endoplasmic reticulum and the autophagy-related signaling pathways, which is consistent with the enrichment results for the differential genes between m6A patterns. This suggests that these genes may be closely related to mitochondrial autophagy, which is consistent with a study that showed m6A mRNA methylation could control autophagy by targeting Atg5 and Atg7 [41]. Therefore, the top 10 genes in the blue synthesis module may provide potential targets for exploring the mechanisms related to autophagy regulation by m6A modification.

However, this study has some shortcomings that need to be clarified. These results are based on bioinformatics analysis; many are theoretical and have not been experimentally validated, so their accuracy needs improvement. Additional genetic engineering methods and immunodeficient mice can be used to reasonably further explore and analyze these computational inferences. However, combining consistent results from multiple bioinformatics analyses and related literature reviews, we propose that these computational predictions can provide a valuable reference for understanding the m6A-related mechanisms of pSS development. Nevertheless, experiments are the optimal criteria for verifying a hypothesis, so we will follow up with further validation through experiments.

## 5. Conclusion

The results of our bioinformatics analysis reveal potential regulatory mechanisms of m6A regulators in the immune infiltration of primary Sjögren's syndrome, which may provide new insights into therapeutic approaches for primary Sjögren's syndrome.

## Data Availability

The data used to support the findings of this study have been deposited in the GEO repository (GSE40611 and GSE84844).
